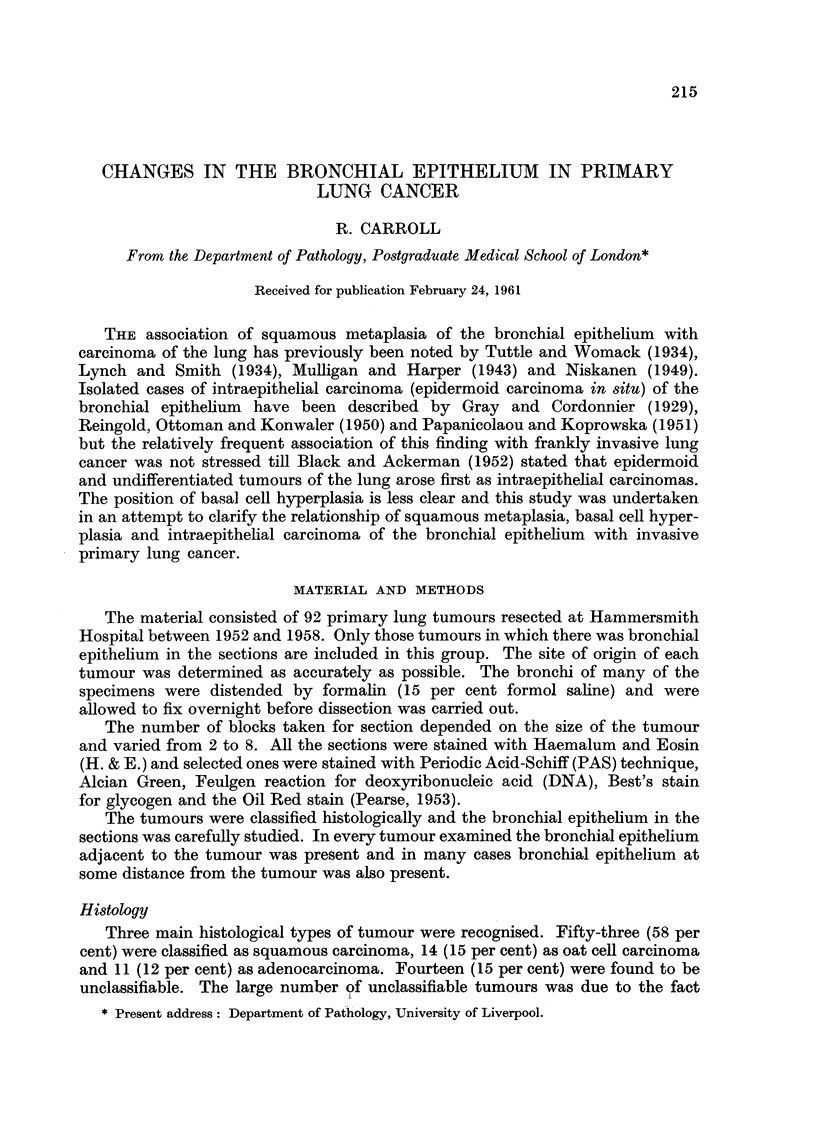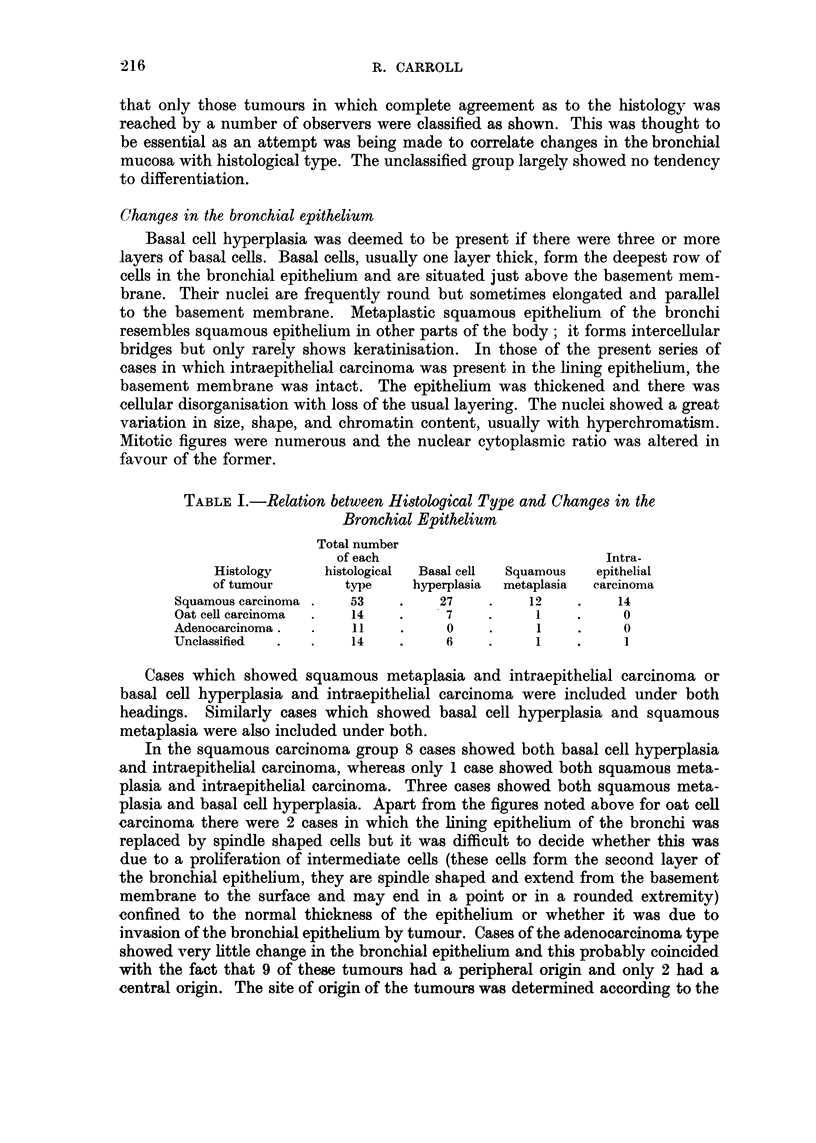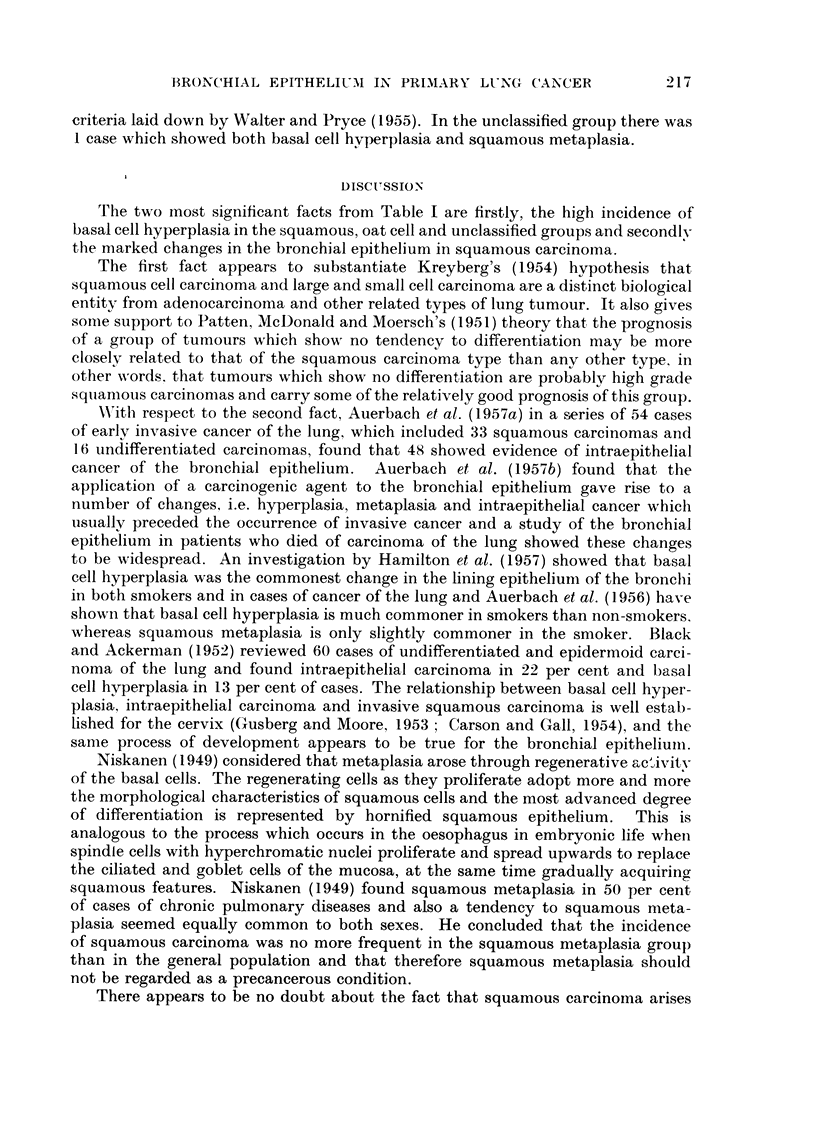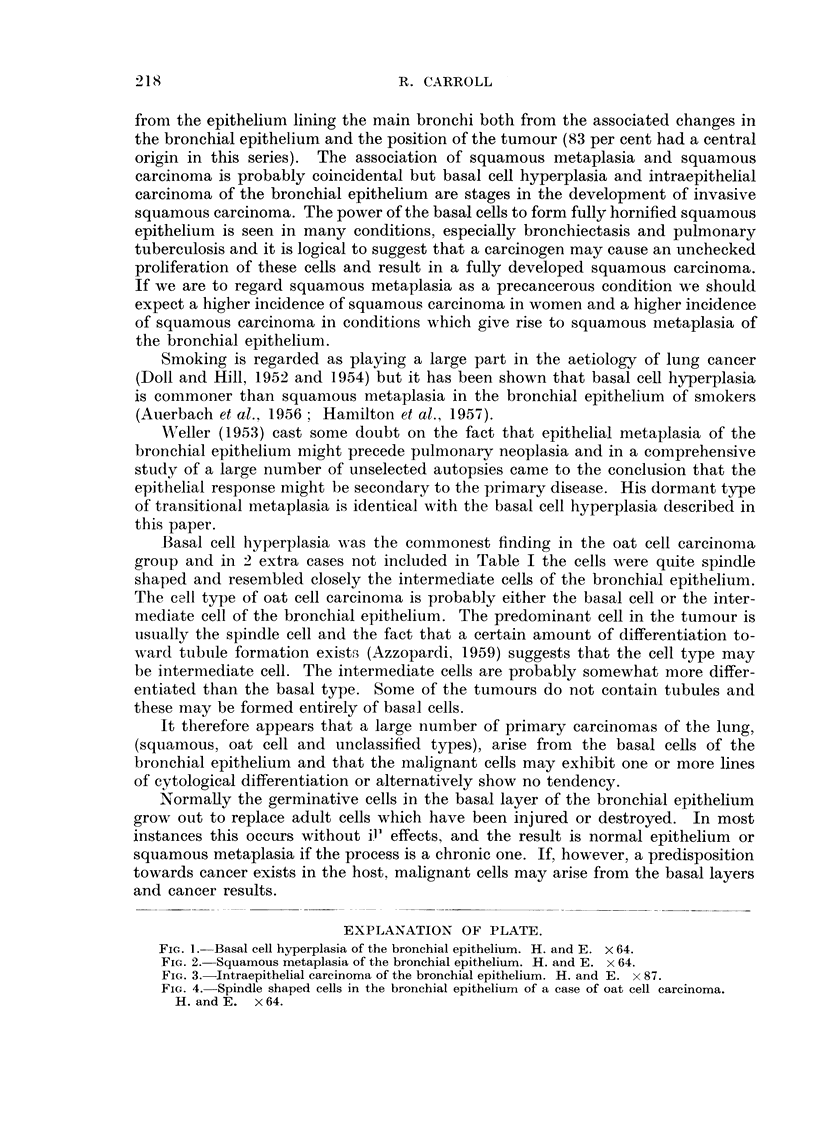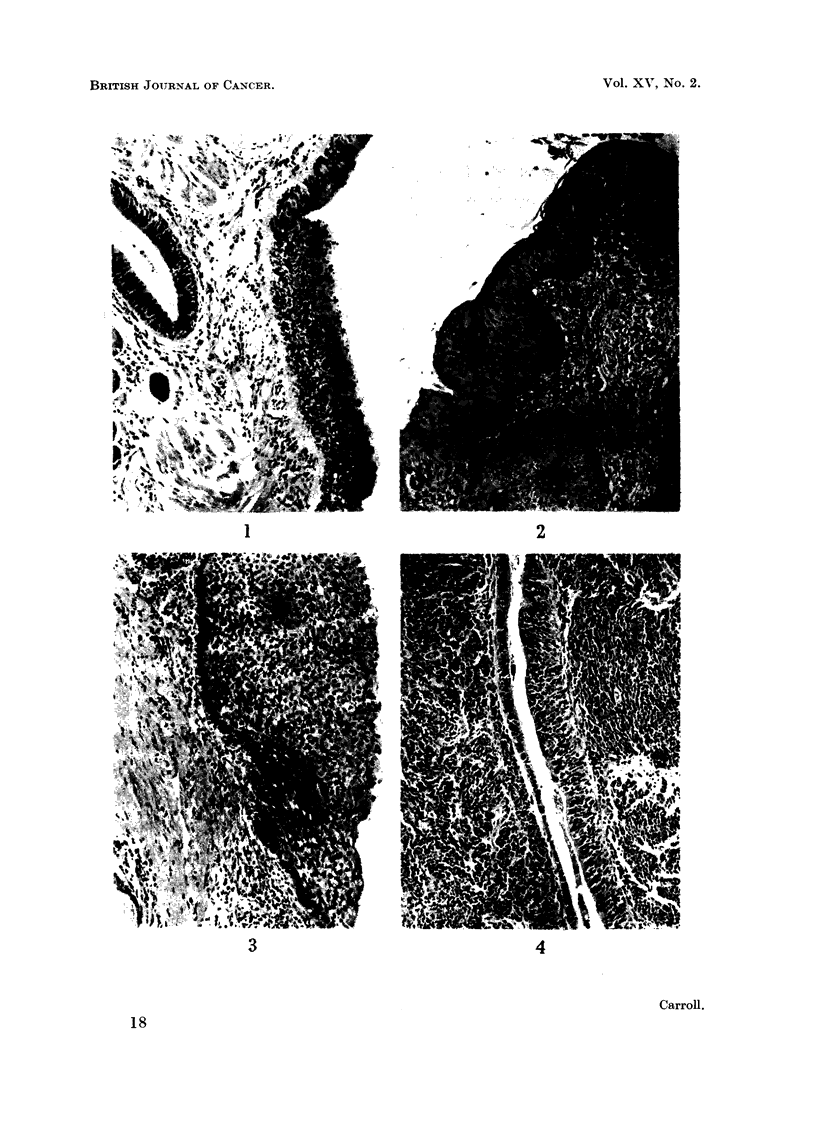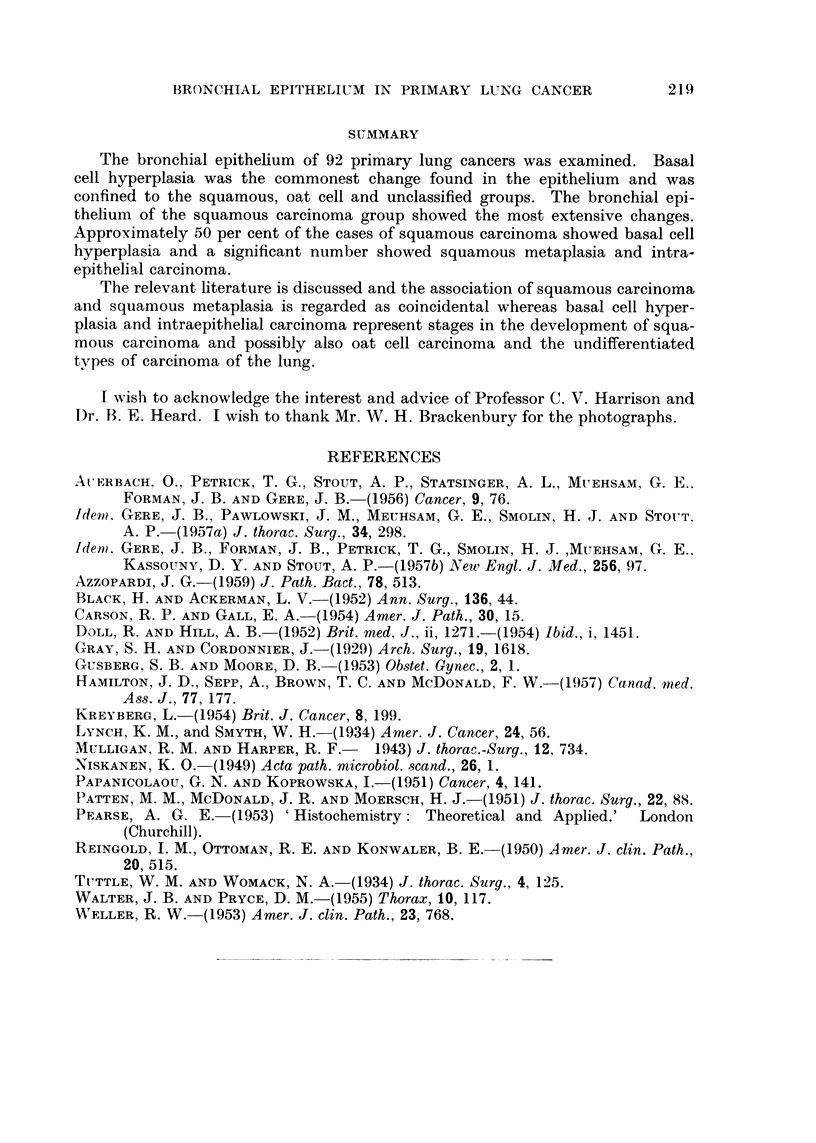# Changes in the Bronchial Epithelium in Primary Lung Cancer

**DOI:** 10.1038/bjc.1961.27

**Published:** 1961-06

**Authors:** R. Carroll

## Abstract

**Images:**


### CHANGES IN THE BRONCHIAL EPITHELIUM IN PRIMARY LUNG CANCER

R. CARROLL

*From the Department of Pathology, Postgraduate Medical School of London**

Received for publication February 24, 1961

THE association of squamous metaplasia of the bronchial epithelium with carcinoma of the lung has previously been noted by Tuttle and Womack (1934), Lynch and Smith (1934), Mulligan and Harper (1943) and Niskanen (1949). Isolated cases of intraepithelial carcinoma (epidermoid carcinoma *in situ*) of the bronchial epithelium have been described by Gray and Cordonnier (1929), Reingold, Ottoman and Konwaler (1950) and Papanicolaou and Koprowska (1951) but the relatively frequent association of this finding with frankly invasive lung cancer was not stressed till Black and Ackerman (1952) stated that epidermoid and undifferentiated tumours of the lung arose first as intraepithelial carcinomas. The position of basal cell hyperplasia is less clear and this study was undertaken in an attempt to clarify the relationship of squamous metaplasia, basal cell hyperplasia and intraepithelial carcinoma of the bronchial epithelium with invasive primary lung cancer.

### MATERIAL AND METHODS

The material consisted of 92 primary lung tumours resected at Hammersmith Hospital between 1952 and 1958. Only those tumours in which there was bronchial epithelium in the sections are included in this group. The site of origin of each tumour was determined as accurately as possible. The bronchi of many of the specimens were distended by formalin (15 per cent formol saline) and were allowed to fix overnight before dissection was carried out.

The number of blocks taken for section depended on the size of the tumour and varied from 2 to 8. All the sections were stained with Haemalum and Eosin (H. & E.) and selected ones were stained with Periodic Acid-Schiff (PAS) technique, Alcian Green, Feulgen reaction for deoxyribonucleic acid (DNA), Best's stain for glycogen and the Oil Red stain (Pearse, 1953).

The tumours were classified histologically and the bronchial epithelium in the sections was carefully studied. In every tumour examined the bronchial epithelium adjacent to the tumour was present and in many cases bronchial epithelium at some distance from the tumour was also present.

### *Histology*

Three main histological types of tumour were recognised. Fifty-three (58 per cent) were classified as squamous carcinoma, 14 (15 per cent) as oat cell carcinoma and 11 (12 per cent) as adenocarcinoma. Fourteen (15 per cent) were found to be unclassifiable. The large number of unclassifiable tumours was due to the fact

* Present address : Department of Pathology, University of Liverpool.

that only those tumours in which complete agreement as to the histology was reached by a number of observers were classified as shown. This was thought to be essential as an attempt was being made to correlate changes in the bronchial mucosa with histological type. The unclassified group largely showed no tendency to differentiation.

*Changes in the bronchial epithelium*

Basal cell hyperplasia was deemed to be present if there were three or more layers of basal cells. Basal cells, usually one layer thick, form the deepest row of cells in the bronchial epithelium and are situated just above the basement membrane. Their nuclei are frequently round but sometimes elongated and parallel to the basement membrane. Metaplastic squamous epithelium of the bronchi resembles squamous epithelium in other parts of the body ; it forms intercellular bridges but only rarely shows keratinisation. In those of the present series of cases in which intraepithelial carcinoma was present in the lining epithelium, the basement membrane was intact. The epithelium was thickened and there was cellular disorganisation with loss of the usual layering. The nuclei showed a great variation in size, shape, and chromatin content, usually with hyperchromatism. Mitotic figures were numerous and the nuclear cytoplasmic ratio was altered in favour of the former.

TABLE I.—*Relation between Histological Type and Changes in the Bronchial Epithelium*

| Histology of tumour | Total number of each histological type | Basal cell hyperplasia | Squamous metaplasia | Intra-epithelial carcinoma |
|---|---|---|---|---|
| Squamous carcinoma | 53 | 27 | 12 | 14 |
| Oat cell carcinoma | 14 | 7 | 1 | 0 |
| Adenocarcinoma | 11 | 0 | 1 | 0 |
| Unclassified | 14 | 6 | 1 | 1 |

Cases which showed squamous metaplasia and intraepithelial carcinoma or basal cell hyperplasia and intraepithelial carcinoma were included under both headings. Similarly cases which showed basal cell hyperplasia and squamous metaplasia were also included under both.

In the squamous carcinoma group 8 cases showed both basal cell hyperplasia and intraepithelial carcinoma, whereas only 1 case showed both squamous metaplasia and intraepithelial carcinoma. Three cases showed both squamous metaplasia and basal cell hyperplasia. Apart from the figures noted above for oat cell carcinoma there were 2 cases in which the lining epithelium of the bronchi was replaced by spindle shaped cells but it was difficult to decide whether this was due to a proliferation of intermediate cells (these cells form the second layer of the bronchial epithelium, they are spindle shaped and extend from the basement membrane to the surface and may end in a point or in a rounded extremity) confined to the normal thickness of the epithelium or whether it was due to invasion of the bronchial epithelium by tumour. Cases of the adenocarcinoma type showed very little change in the bronchial epithelium and this probably coincided with the fact that 9 of these tumours had a peripheral origin and only 2 had a central origin. The site of origin of the tumours was determined according to the criteria laid down by Walter and Pryce (1955). In the unclassified group there was 1 case which showed both basal cell hyperplasia and squamous metaplasia.

### DISCUSSION

The two most significant facts from Table I are firstly, the high incidence of basal cell hyperplasia in the squamous, oat cell and unclassified groups and secondly the marked changes in the bronchial epithelium in squamous carcinoma.

The first fact appears to substantiate Kreyberg's (1954) hypothesis that squamous cell carcinoma and large and small cell carcinoma are a distinct biological entity from adenocarcinoma and other related types of lung tumour. It also gives some support to Patten, McDonald and Moersch's (1951) theory that the prognosis of a group of tumours which show no tendency to differentiation may be more closely related to that of the squamous carcinoma type than any other type, in other words, that tumours which show no differentiation are probably high grade squamous carcinomas and carry some of the relatively good prognosis of this group.

With respect to the second fact, Auerbach *et al.* (1957*a*) in a series of 54 cases of early invasive cancer of the lung, which included 33 squamous carcinomas and 16 undifferentiated carcinomas, found that 48 showed evidence of intraepithelial cancer of the bronchial epithelium. Auerbach *et al.* (1957*b*) found that the application of a carcinogenic agent to the bronchial epithelium gave rise to a number of changes, i.e. hyperplasia, metaplasia and intraepithelial cancer which usually preceded the occurrence of invasive cancer and a study of the bronchial epithelium in patients who died of carcinoma of the lung showed these changes to be widespread. An investigation by Hamilton *et al.* (1957) showed that basal cell hyperplasia was the commonest change in the lining epithelium of the bronchi in both smokers and in cases of cancer of the lung and Auerbach *et al.* (1956) have shown that basal cell hyperplasia is much commoner in smokers than non-smokers, whereas squamous metaplasia is only slightly commoner in the smoker. Black and Ackerman (1952) reviewed 60 cases of undifferentiated and epidermoid carcinoma of the lung and found intraepithelial carcinoma in 22 per cent and basal cell hyperplasia in 13 per cent of cases. The relationship between basal cell hyperplasia, intraepithelial carcinoma and invasive squamous carcinoma is well established for the cervix (Gusberg and Moore, 1953 ; Carson and Gall, 1954), and the same process of development appears to be true for the bronchial epithelium.

Niskanen (1949) considered that metaplasia arose through regenerative activity of the basal cells. The regenerating cells as they proliferate adopt more and more the morphological characteristics of squamous cells and the most advanced degree of differentiation is represented by hornified squamous epithelium. This is analogous to the process which occurs in the oesophagus in embryonic life when spindle cells with hyperchromatic nuclei proliferate and spread upwards to replace the ciliated and goblet cells of the mucosa, at the same time gradually acquiring squamous features. Niskanen (1949) found squamous metaplasia in 50 per cent of cases of chronic pulmonary diseases and also a tendency to squamous metaplasia seemed equally common to both sexes. He concluded that the incidence of squamous carcinoma was no more frequent in the squamous metaplasia group than in the general population and that therefore squamous metaplasia should not be regarded as a precancerous condition.

There appears to be no doubt about the fact that squamous carcinoma arises from the epithelium lining the main bronchi both from the associated changes in the bronchial epithelium and the position of the tumour (83 per cent had a central origin in this series). The association of squamous metaplasia and squamous carcinoma is probably coincidental but basal cell hyperplasia and intraepithelial carcinoma of the bronchial epithelium are stages in the development of invasive squamous carcinoma. The power of the basal cells to form fully hornified squamous epithelium is seen in many conditions, especially bronchiectasis and pulmonary tuberculosis and it is logical to suggest that a carcinogen may cause an unchecked proliferation of these cells and result in a fully developed squamous carcinoma. If we are to regard squamous metaplasia as a precancerous condition we should expect a higher incidence of squamous carcinoma in women and a higher incidence of squamous carcinoma in conditions which give rise to squamous metaplasia of the bronchial epithelium.

Smoking is regarded as playing a large part in the aetiology of lung cancer (Doll and Hill, 1952 and 1954) but it has been shown that basal cell hyperplasia is commoner than squamous metaplasia in the bronchial epithelium of smokers (Auerbach *et al.*, 1956 ; Hamilton *et al.*, 1957).

Weller (1953) cast some doubt on the fact that epithelial metaplasia of the bronchial epithelium might precede pulmonary neoplasia and in a comprehensive study of a large number of unselected autopsies came to the conclusion that the epithelial response might be secondary to the primary disease. His dormant type of transitional metaplasia is identical with the basal cell hyperplasia described in this paper.

Basal cell hyperplasia was the commonest finding in the oat cell carcinoma group and in 2 extra cases not included in Table I the cells were quite spindle shaped and resembled closely the intermediate cells of the bronchial epithelium. The cell type of oat cell carcinoma is probably either the basal cell or the intermediate cell of the bronchial epithelium. The predominant cell in the tumour is usually the spindle cell and the fact that a certain amount of differentiation toward tubule formation exists (Azzopardi, 1959) suggests that the cell type may be intermediate cell. The intermediate cells are probably somewhat more differentiated than the basal type. Some of the tumours do not contain tubules and these may be formed entirely of basal cells.

It therefore appears that a large number of primary carcinomas of the lung, (squamous, oat cell and unclassified types), arise from the basal cells of the bronchial epithelium and that the malignant cells may exhibit one or more lines of cytological differentiation or alternatively show no tendency.

Normally the germinative cells in the basal layer of the bronchial epithelium grow out to replace adult cells which have been injured or destroyed. In most instances this occurs without ill effects, and the result is normal epithelium or squamous metaplasia if the process is a chronic one. If, however, a predisposition towards cancer exists in the host, malignant cells may arise from the basal layers and cancer results.

### EXPLANATION OF PLATE.

FIG. 1.—Basal cell hyperplasia of the bronchial epithelium. H. and E. ×64.
FIG. 2.—Squamous metaplasia of the bronchial epithelium. H. and E. ×64.
FIG. 3.—Intraepithelial carcinoma of the bronchial epithelium. H. and E. ×87.
FIG. 4.—Spindle shaped cells in the bronchial epithelium of a case of oat cell carcinoma. H. and E. ×64.

1

2

3

4

Carroll.

SUMMARY

The bronchial epithelium of 92 primary lung cancers was examined. Basal cell hyperplasia was the commonest change found in the epithelium and was confined to the squamous, oat cell and unclassified groups. The bronchial epithelium of the squamous carcinoma group showed the most extensive changes. Approximately 50 per cent of the cases of squamous carcinoma showed basal cell hyperplasia and a significant number showed squamous metaplasia and intraepithelial carcinoma.

The relevant literature is discussed and the association of squamous carcinoma and squamous metaplasia is regarded as coincidental whereas basal cell hyperplasia and intraepithelial carcinoma represent stages in the development of squamous carcinoma and possibly also oat cell carcinoma and the undifferentiated types of carcinoma of the lung.

I wish to acknowledge the interest and advice of Professor C. V. Harrison and Dr. B. E. Heard. I wish to thank Mr. W. H. Brackenbury for the photographs.

REFERENCES

AUERBACH, O., PETRICK, T. G., STOUT, A. P., STATSINGER, A. L., MUEHSAM, G. E., FORMAN, J. B. AND GERE, J. B.—(1956) *Cancer*, **9**, 76.

*Idem*. GERE, J. B., PAWLOWSKI, J. M., MEUHSAM, G. E., SMOLIN, H. J. AND STOUT, A. P.—(1957*a*) *J. thorac. Surg.*, **34**, 298.

*Idem*. GERE, J. B., FORMAN, J. B., PETRICK, T. G., SMOLIN, H. J. ,MUEHSAM, G. E., KASSOUNY, D. Y. AND STOUT, A. P.—(1957*b*) *New Engl. J. Med.*, **256**, 97.

AZZOPARDI, J. G.—(1959) *J. Path. Bact.*, **78**, 513.

BLACK, H. AND ACKERMAN, L. V.—(1952) *Ann. Surg.*, **136**, 44.

CARSON, R. P. AND GALL, E. A.—(1954) *Amer. J. Path.*, **30**, 15.

DOLL, R. AND HILL, A. B.—(1952) *Brit. med. J.*, ii, 1271.—(1954) *Ibid.*, i, 1451.

GRAY, S. H. AND CORDONNIER, J.—(1929) *Arch. Surg.*, **19**, 1618.

GUSBERG, S. B. AND MOORE, D. B.—(1953) *Obstet. Gynec.*, **2**, 1.

HAMILTON, J. D., SEPP, A., BROWN, T. C. AND MCDONALD, F. W.—(1957) *Canad. med. Ass. J.*, **77**, 177.

KREYBERG, L.—(1954) *Brit. J. Cancer*, **8**, 199.

LYNCH, K. M., and SMYTH, W. H.—(1934) *Amer. J. Cancer*, **24**, 56.

MULLIGAN, R. M. AND HARPER, R. F.— 1943) *J. thorac.-Surg.*, **12**, 734.

NISKANEN, K. O.—(1949) *Acta path. microbiol. scand.*, **26**, 1.

PAPANICOLAOU, G. N. AND KOPROWSKA, I.—(1951) *Cancer*, **4**, 141.

PATTEN, M. M., MCDONALD, J. R. AND MOERSCH, H. J.—(1951) *J. thorac. Surg.*, **22**, 88.

PEARSE, A. G. E.—(1953) 'Histochemistry: Theoretical and Applied.' London (Churchill).

REINGOLD, I. M., OTTOMAN, R. E. AND KONWALER, B. E.—(1950) *Amer. J. clin. Path.*, **20**, 515.

TUTTLE, W. M. AND WOMACK, N. A.—(1934) *J. thorac. Surg.*, **4**, 125.

WALTER, J. B. AND PRYCE, D. M.—(1955) *Thorax*, **10**, 117.

WELLER, R. W.—(1953) *Amer. J. clin. Path.*, **23**, 768.